# From stage to screen: performance language mediation and audience engagement in Chinese and Anglo-American musical theater film adaptations

**DOI:** 10.3389/fpsyg.2026.1828417

**Published:** 2026-05-15

**Authors:** Qin Lin

**Affiliations:** 1 University of Nottingham, Nottingham, United Kingdom; 2 Future Imaging Laboratory, Innovation Center of Yangtze River Delta, Zhejiang University, Jiaxing, Zhejiang, China

**Keywords:** audience experiment, CASS model, cross-cultural reception, musical film, performance adaptation, screen language, stage language

## Abstract

Adapting musical theater into film involves the mediation of performance language from stage to screen. While stage language depends on live singing, spatial ensemble, and performer–audience co-presence, screen language relies on framing, editing, and audiovisual control. Although musical theater film adaptation has attracted increasing scholarly attention, audience-based research on how this transformation is perceived remains limited, especially in Chinese and Anglo-American contexts. This study examines how stage language is transformed into screen language through four stage-to-screen cases: the musical theater Les Misérables, first staged in 1980, and its 2012 film adaptation; the musical theater Dear Evan Hansen, first staged in 2017, and its 2021 film adaptation; the original Chinese musical theater Jinsha, first staged in 2005, and its 2023 film adaptation; and the original Chinese musical theater The Long Night, first performed in 2021, and its 2022 screen version. Guided by the Cultural Adaptability Symbolic Stratification (CASS) model, the study adopts a mixed-methods approach combining case analysis, content analysis, questionnaires, and focus group discussions with 27 participants. The findings identify three pathways of transformation: Cinematic Translation, Direct Transplantation, and Fragmented Reconfiguration. Among them, Cinematic Translation generated the strongest audience engagement, especially in emotional immersion and narrative clarity. Direct Transplantation preserved visible stage conventions but often weakened narrative and emotional coherence. Fragmented Reconfiguration produced more variable responses, depending on viewers’ interpretive expectations and familiarity with non-linear form. Some age-related tendencies also appeared within this sample, but these should be treated cautiously given the limited number of middle-aged and older participants. Overall, the study shows that successful adaptation depends less on preserving theatrical form than on cinematically reorganizing performance language. It also refines the application of the CASS model to performance analysis and offers practical insight into the adaptation of Chinese original musical theater works into musical theater film.

## 1 Introduction

### 1.1 Research background

Musical theater adaptation into film is one of the most complex forms of cross-media storytelling because it requires the reorganization of performance across different media. A musical theater film is defined as a genre that incorporates substantial song and dance segments, using singing to characterize inner worlds and advance the plot (Lin, 2022). At the center of this process lies the tension between stage language and screen language. Stage language depends on live immediacy, spatial ensemble, and performer–audience co-presence, whereas screen language is shaped by framing, editing, and audiovisual control (Horovitz, 2004; Gorbman, 1980; Metz, 1982). When a musical theater work is adapted into film, performance cannot simply be transferred from one medium to another. It must be reformulated.

This tension is evident in two widely discussed Anglo-American cases *(Les Misérables and Dear Evan Hansen*), which are examined in detail in Section 4. The contrasting outcomes of these adaptations suggest that successful adaptation depends less on fidelity to the source text than on the effective mediation of performance language across media.

This study examines this problem in both Anglo-American and Chinese contexts, with particular attention to the adaptation of Chinese original musical theater into film. The Chinese cases, *Jinsha* (2005 stage, 2023 film) and *The Long Night* (2021 stage, 2022 screen version), illuminate the challenges of translating Chinese stories, symbols, and performance traditions into cinematic form. Here, the issue is not simply how to adapt a musical theater work, but how to develop a viable cinematic language for Chinese original musicals.

Despite growing attention to musical theater film adaptation, gaps remain. Adaptation studies have focused on narrative fidelity and medium transfer (Hutcheon, 2006; Stam and Raengo, 2005), and medium-specificity theory on technical differences between theater and film (Barthes, 1977; Metz, 1982). Yet empirical research on audience perception of performance language is limited, especially for Chinese musical theater film, where cultural localisation, symbolic translation, and performance style are underexamined (Li, 2011; Liang, 2023; Duan, 2023).

This study addresses this gap through an audience-centered, performance-focused investigation guided by the CASS model. Using a mixed-methods design including case analysis, content analysis, questionnaires, and focus groups with 27 participants across four cases, it examines how stage language transforms into screen language and how adaptation strategies affect emotional immersion, narrative clarity, and interpretive engagement.

The central argument: successful adaptation depends not on preserving theatrical form, but on cinematically reorganizing performance language. For Chinese original musicals, cultural richness must be restructured into cinematically coherent, narratively persuasive, and emotionally legible forms. The study contributes theoretically by refining the CASS model for performance analysis, empirically by providing audience-based evidence on stage-to-screen mediation, and practically by offering a framework for adapting Chinese stories into compelling musical films.

### 1.2 Research questions

This study investigates how musical theater works are transformed when adapted into film, with particular attention to the mediation of performance language. The research addresses the following questions:

*RQ1*: How is stage language transformed into screen language in the adaptation of musical theater works into film across Chinese and Anglo-American contexts?

This question examines the specific strategies through which stage-derived performance conventions, including live singing, spatial ensemble, and stylised gesture, are reconfigured for cinematic presentation. Particular attention is given to techniques such as shot selection, editing rhythm, and audiovisual integration, and to how these techniques shape continuity between stage performance and screen expression.

*RQ2*: How do different pathways of performance language transformation affect audience emotional immersion and narrative comprehension?

This question investigates the relationship between adaptation strategy and audience response. Drawing on focus group data and questionnaire results, it examines which transformation pathways most effectively generate emotional resonance and narrative clarity, and how these effects vary across participant groups and levels of familiarity with musical theater.

*RQ3*: What cross-media strategies enable the effective mediation of performance language in musical theater film adaptation, and what are the limits of such mediation?

This question explores the boundaries of performance language translation. It examines cases in which stage language resists cinematic integration, identifying factors such as cultural specificity, performance style, and audience expectation that may constrain successful adaptation. It also considers the role of celebrity culture in shaping reception, as well as its limitations in compensating for disjunctions between stage-derived performance and cinematic form.

Taken together, these questions provide a framework for analyzing musical theater adaptation as a negotiation between two distinct performance languages, with measurable consequences for audience engagement and for the adaptation of Chinese original musical theater works into film.

## 2 Literature review

The adaptation of musical theater works into film has attracted substantial scholarly attention, yet important gaps remain in relation to the mediation of performance language and the comparative study of Chinese and Anglo-American practices. Existing research has examined narrative, aesthetic, and performative dimensions of adaptation, but relatively few studies have focused on how these elements are reorganized across media and cultural contexts through the transformation of performance language. This gap is especially evident in relation to Chinese musical theater film, where questions of cultural localisation, symbolic translation, and stage-to-screen performance remain insufficiently explored. This Section reviews key perspectives from adaptation studies, medium-specificity theory, and celebrity culture, and then identifies the theoretical and empirical gaps that the present study addresses through the Cultural Adaptability Symbolic Stratification (CASS) model.

### 2.1 Adaptation studies and the question of performance

Contemporary scholarship has largely moved beyond fidelity-based views of adaptation. Hutcheon (2006) regards adaptation as a creative reinterpretation across media and cultures, emphasizing that adaptations are autonomous works that engage with their sources in a dialogic rather than derivative way. Stam and Raengo (2005) frames adaptation as a process shaped by broader cultural and discursive systems, where meaning is continually renegotiated rather than simply transferred. These theoretical advances have illuminated adaptation as transformation, yet they rarely address how performance itself is reconfigured across media. Most research emphasizes narrative structure and thematic continuity without systematically examining how singing, gesture, and spatial presence are translated from stage to screen.

This gap is particularly significant for musical theater, where performance is not merely a vehicle for narrative but constitutes the primary medium of emotional expression. When a stage musical is adapted into film, the performer’s body, voice, and relationship to the audience space undergo a fundamental transformation. Adaptation studies have yet to develop analytical tools capable of tracking these changes at the level of performance language. This study addresses this gap by applying the CASS model to analyze how performance operates across surface, structural, and deep symbolic levels, particularly in comparative analysis of Chinese and Anglo-American adaptations.

### 2.2 Medium-specificity theory and performance language

Medium-specificity theory provides essential groundwork for understanding the differences between stage and screen performance. Barthes (1977) and Metz (1982) emphasize the distinct expressive capacities of theater and film, arguing that each medium possesses unique semiotic systems that shape how meaning is produced and perceived. Musical theater relies on live performance, spatial immediacy, and real-time audience interaction, creating meaning through the co-presence of performers and spectators. Cinema, in contrast, employs framing, editing, sound design, and post-production manipulation to construct meaning through carefully controlled audiovisual compositions (Gorbman, 1980).

Existing work has thoroughly described these technical differences but has paid less attention to their implications for performance language. The transition from live singing to recorded and edited vocal performance, from continuous stage blocking to fragmented shot coverage, and from ensemble choreography to close-up gestures all represent transformations that fundamentally alter how performance signifies. Moreover, the cultural implications of these sensory transformations remain underexplored. How do audiences conditioned by different performance traditions perceive these shifts? What happens when Anglo-American-derived stage conventions are adapted into Chinese cinematic contexts? This study investigates how such sensory transformations alter symbolic meaning and audience reception, particularly in cross-cultural contexts where expectations of performance differ significantly.

### 2.3 Celebrity culture and performance reception

Celebrity culture plays an increasingly significant role in shaping audience reception of musical theater film adaptations. Jenkins (2006) highlights how convergence culture and celebrity branding shape audience engagement, with stars functioning as transmedia commodities whose personas circulate across platforms and influence interpretation. In China, the rise of idol culture and variety shows has further complicated this dynamic, blurring the line between artistic integrity and commercial spectacle (Liu, 2024). Programs such as *Super Vocal* and *PhantaCity* have created new pathways for musical theater exposure while simultaneously foregrounding celebrity personas over performance content.

While celebrity casting can expand visibility and attract diverse audiences, it may also weaken narrative coherence and symbolic depth when star image conflicts with character demands or when performance is subordinated to persona. Anglo-American scholarship has not fully considered these dynamics in Chinese musical adaptations, where the idol economy creates distinctive conditions for performance reception. This study addresses this gap by analyzing how star-driven strategies reshape performance, language translation, and audience interpretation, examining cases where celebrity presence either enhances or disrupts the mediation of stage language into screen language.

### 2.4 Research gaps and contributions

The literature reviewed above reveals three interconnected gaps that this study aims to address. First, adaptation studies have insufficiently theorized performance language as a distinct object of analysis. While narrative and thematic transformation have received extensive attention, the question of how singing, gesture, and spatial presence are reconfigured across media remains under-examined. This study develops analytical tools for tracking performance language transformation through the CASS model. Second, medium-specificity theory has described technical differences between theater and film but has not systematically investigated how these differences affect audience perception of performance, particularly across cultural contexts. This study provides empirical data on how audiences perceive and evaluate different strategies for translating stage language into screen language. Third, celebrity culture research has examined how star power shapes reception but has not addressed how celebrity performance itself is transformed across media or how it interacts with performance language mediation. This study examines the conditions under which celebrity presence facilitates or hinders the successful translation of performance.

By applying the CASS model to comparative analysis of Chinese and Anglo-American musical theater film adaptations, this study offers a framework for understanding performance language transformation that integrates insights from adaptation theory, medium-specificity theory, and celebrity culture studies. It contributes theoretically by refining the CASS model for performance analysis, empirically by providing comparative audience data across cultural contexts, and practically by identifying strategies that enhance or impede successful performance mediation.

The next Section presents the methodology, outlining the research design and approach used to investigate these questions through case analysis, content analysis, and audience experiments.

## 3 Methodology and theoretical framework

Building on the theoretical foundation established in the literature review, this Section outlines the research methodology and theoretical framework guiding the study. The approach combines both qualitative and quantitative methods to provide a comprehensive analysis of how performance language is mediated in musical theater adaptations into film, with particular focus on Chinese and Anglo-American contexts.

### 3.1 Methodology

#### 3.1.1 Research design

This study employs an embedded mixed-methods design integrating textual analysis with audience research. The design addresses three dimensions aligned with the research questions: narrative transformation, performance language mediation, and audience reception. Case analysis and content analysis establish the comparative foundation, while video-based viewing tests and focus group discussions provide experimental data on audience response. This combination enables analysis of both structural transformations in adaptation and the lived responses of viewers.

#### 3.1.2 Data collection and primary sources

##### 3.1.2.1 Case analysis

This study compares selected Chinese and Anglo-American musical theater works and their screen adaptations. To provide clear geographical context: the stage original of *Les Misérables* refers to the West End production (United Kingdom); *Dear Evan Hansen* refers to the Broadway production (United States); and the supplementary reference *The Phantom of the Opera* refers to the work by Andrew Lloyd Webber (United Kingdom).

The four focal cases are *Les Misérables, Dear Evan Hansen, Jinsha*, and *The Long Night*, while *The Phantom Lover* serve as supplementary comparative reference. The Phantom Lover is included because its screen adaptation partially draws on Lloyd Webber’s *The Phantom of the Opera*, offering a glimpse into how a Chinese musical film reworks a Western stage source. In this study, *The Long Night* refers to the screen adaptation of the musical theater work rather than the 2020 television series of the same title. The analysis focuses on narrative transformation, medium-specific adaptation strategies, cultural localisation, and the role of celebrity casting.

##### 3.1.2.2 Content analysis

Content analysis is used to evaluate shifts in expressive strategies across stage and screen versions. The analysis focuses on four variables:

1. Narrative structure, including plot condensation and scene reordering.
2. Musical arrangement and stylistic change, including orchestration and tempo.
3. Performance language, including acting style, vocal technique, and gesture.
4. Cinematic language, including cinematography, editing, mise-en-scène, and audiovisual synchronization.

##### 3.1.2.3 Focus group design and data collection

To explore audience perceptions of performance language transformation, the study used controlled video viewing followed by focus group interviews. A total of 27 participants (aged 12–60) were recruited across five groups, organized by age range and self-reported familiarity with musical theater to ensure varied prior exposure. Most participants were based in China; linguistic competence and intercultural experience were not measured formally, a limitation that should be considered when interpreting the reception findings. The age distribution was uneven (18–30 age group: 18 participants; over 50: 4 participants; 31–50: 2 participants), which limits generalisability.

Each 90-min session included 30 min of viewing and 60 min of moderated discussion. Participants watched curated excerpts from the selected Chinese and Anglo-American screen cases alongside their stage counterparts. Non-Chinese materials were shown with Chinese subtitles to support comprehension.

After each excerpt, participants completed a 5-point Likert questionnaire measuring emotional immersion, narrative clarity, cultural recognition, and aesthetic satisfaction. This was followed by a semi-structured discussion guided by open-ended prompts on symbolic interpretation, audiovisual preference, perceived authenticity, and adaptation logic. Sessions were audio-recorded, transcribed verbatim, and coded inductively in NVivo to identify recurring themes (e.g., authenticity, immersion, clarity). This design captured both immediate quantitative responses and reflective qualitative commentary.

#### 3.1.3 Data analysis

Data analysis combines several methods. Narrative and thematic analysis trace changes in narrative structure, characterisation and thematic emphasis through close reading of the selected texts. Semiotic analysis, informed by Barthes (1977) and Metz (1982), examines shifts in visual, musical, and performative signs. The Cultural Adaptability Symbolic Stratification (CASS) model serves as the primary interpretive framework, with its three levels of surface symbols, structural symbols, and deep cultural symbols guiding analysis of how performance language is retained, altered, or lost in adaptation.

Reception analysis combines inductive coding of focus group transcripts with sentiment analysis of user-generated content from Douban, Xiaohongshu, and Weibo. Chinese-language processing tools, including Jieba and SnowNLP, are used to extract keywords and emotional polarity from online discourse. Descriptive statistics summaries Likert-scale responses, while exploratory statistical comparisons are used to examine differences across participant groups, especially in relation to age and theater or film orientation. Because the online discourse data are drawn primarily from Chinese-language platforms, this part of the analysis should not be understood as a fully symmetrical cross-cultural comparison.

#### 3.1.4 Ethics approval

Ethical approval for this study was obtained from a university ethics review board prior to data collection. Participants received clear information about the aims and procedures of the study and provided written informed consent before taking part. All personal identifiers were removed during transcription and analysis, and pseudonyms were used where necessary to ensure confidentiality. The data were stored securely and used solely for academic research purposes.

### 3.2 Theoretical framework

This study draws on three interrelated theoretical perspectives to examine how musical theater works are transformed in the process of stage-to-screen adaptation. Together, these perspectives make it possible to analyze adaptation not only as a change of medium, but also as a reorganization of performance language, symbolic meaning, and audience engagement.

#### 3.2.1 Adaptation theory

Adaptation theory has evolved from fidelity-based models toward a more dialogic understanding of creative reinterpretation (Hutcheon, 2006; Stam and Raengo, 2005). Rather than treating adaptation as a secondary or derivative form, this perspective understands it as a process of transformation in which meaning is reworked across media, contexts, and audiences. In musical theater film adaptation, adaptation theory is particularly useful for explaining how narrative structure, thematic emphasis, and symbolic meaning are reshaped when stage language is translated into screen language.

#### 3.2.2 Medium-specificity theory

Medium-specificity theory (Barthes, 1977; Metz, 1982) examines how each medium possesses distinctive expressive capacities. Theater’s live spatial immediacy contrasts with film’s ability to manipulate time and space through close-ups, editing, and visual framing. This study draws on the theory to analyze how filmmakers navigate the tension between theatrical performance and cinematic expression.

#### 3.2.3 Celebrity culture and popular media

Celebrity culture shapes audience engagement with musical theater film adaptations (Jenkins, 2006). In China, the rise of variety shows and the idol economy has increased the prominence of celebrity casting (Liu, 2024). This study examines how celebrity influence both enhances and challenges the reception of performance language, particularly when balancing popular appeal with narrative coherence.

#### 3.2.4 Cultural adaptability symbolic stratification model (CASS)

**FIGURE 1:**
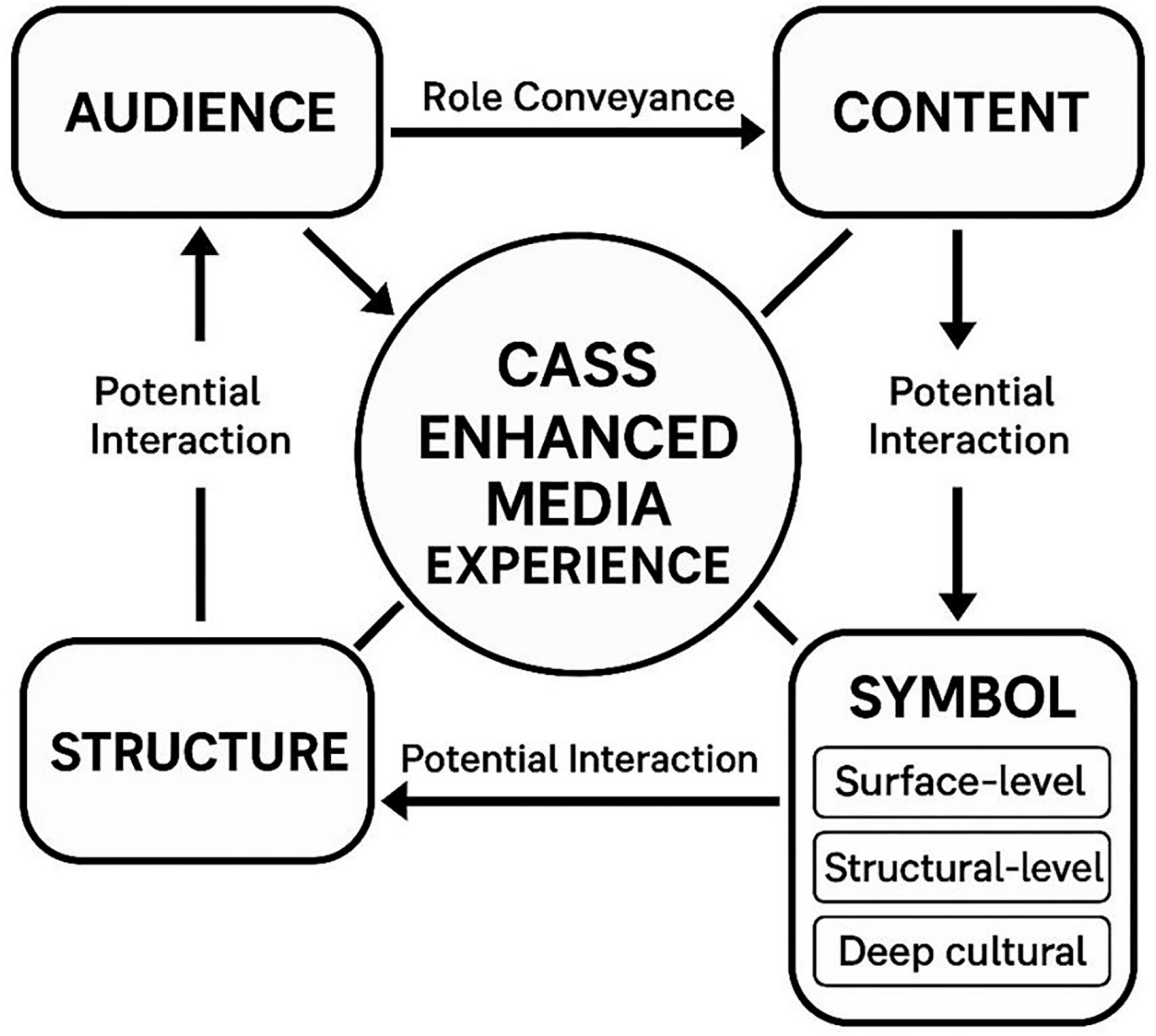
CASS model diagram.

The CASS model is the central analytical tool of this study. As illustrated in Figure 1, the model organizes symbolic elements into three hierarchical levels. At the surface level, symbols are directly perceivable, including costume, setting, music style, and spoken language. At the structural level, symbols are embedded in plot design, character relationships, and modes of conflict resolution. At a deep level, symbols convey abstract values and ideologies, such as collectivism versus individualism or national identity.

This framework enables systematic tracking of how performance language is retained, reinterpreted, or lost during adaptation. The model’s research value lies in its capacity to trace how symbols are preserved, reinterpreted, or omitted in cross-media adaptations. It also highlights potential mismatches between cultural intention and audience reception, offering insights into the opportunities and risks of cultural localisation and globalization in musical theater film adaptations. By identifying symbolic disjunction when cultural meanings are not effectively re-encoded for cinematic or cross-cultural comprehension, the CASS model provides a diagnostic tool for assessing adaptation effectiveness.

#### 3.2.5 Limitations of the theoretical framework

While the CASS model provides a comprehensive lens, it has limitations. Its hierarchical view may not fully capture the fluidity of cultural exchange, and the subjective nature of symbolic interpretation complicates measurement. Moreover, the model assumes a relatively static view of cultural symbols, which may not account for their evolution in globalized media environments.

### 3.3 Research questions and analytical expectations

Based on the theoretical framework, the following analytical expectations guide the investigation:

*E1*: Chinese and Anglo-American cases will likely show different patterns in transforming narrative structure, performance language, and audiovisual form during stage-to-screen adaptation.

*E2*: For Chinese original musicals, cultural localisation alone does not guarantee stronger emotional resonance or clearer symbolic meaning; effectiveness depends on how fully culturally specific elements are integrated into cinematic narrative and performance form.

*E3*: Adaptation strategies that achieve stronger emotional coherence, symbolic clarity, and cinematic integration are more likely to generate higher audience engagement.

## 4 Findings and discussion

This Section presents the findings derived from the case analysis, content analysis, and audience research. Following the presentation of these findings, an in-depth discussion of their implications is provided. By integrating both qualitative and quantitative data, the study sheds light on how narrative structures, aesthetic choices, and cultural symbols are transformed during the adaptation of Chinese and Anglo-American musical theater works into film. The findings are organized according to the core dimensions of the Cultural Adaptability Symbolic Stratification (CASS) model: surface-level, structural, and deep cultural symbols. These findings highlight the pathways that lead to audience resonance or symbolic disjunction, offering insights into how performance language is mediated in cross-cultural contexts.

### 4.1 Findings

The following section outlines the key findings from the case analysis and audience research, identifying three distinct pathways of performance language transformation. These pathways reflect the varied ways in which stage language is restructured into screen language across different adaptation strategies.

#### 4.1.1 Case analysis results: three pathways of performance language transformation

Analysis of the four case studies reveals three distinct pathways for transforming stage language into screen language, each validated through audience research. These pathways correspond to the levels of surface, structural, and deep cultural symbols as outlined in the CASS model.

1. *Cinematic translation*: This pathway involves reconfiguring stage performance elements using cinematic techniques while maintaining their emotional essence. In *Les Misérables (2012)*, live singing is captured through close-ups, intensifying the emotional connection between the characters and the audience. This transformation preserves surface-level symbols (e.g., vocal style), structural symbols (e.g., the redemption arc), and deep cultural symbols (e.g., sacrifice), making them accessible to a global audience.
2. *Direct transplantation*: In this pathway, stage conventions are largely retained but not fully integrated into cinematic language. In *Jinsha (2023)*, extended musical sequences disrupt the flow of the film, creating what participants described as *“performance islands.”* While surface symbols (e.g., costumes) remain strong, the lack of integration leads to symbolic overload without advancing the narrative, diminishing emotional engagement.
3. *Fragmented reconfiguration:* In The Long Night (2021), stage performance elements are fragmented and embedded within a cinematic narrative structure. This non-linear approach encourages active interpretive engagement from the audience. While surface and structural symbols are effectively reconfigured, the success of this pathway depends on the audience’s familiarity with both theatrical and cinematic conventions.

These pathways illustrate how performance language can be adapted across media, balancing the emotional immediacy of stage performance with the expressive possibilities of cinema.

##### 4.1.1.1 Pathway 1: cinematic translation of stage language

***Les misérables (2012)***

*Les Misérables (2012)* exemplifies Cinematic Translation. The most prominent strategy is the use of live singing during filming, combined with close-up framing and sustained shots. This method retains the immediacy characteristic of stage language while adapting it for the screen, making that immediacy legible within the cinematic form.

Although the film broadly follows the narrative structure of the stage version, its emotional impact is heightened through medium-specific devices. In key numbers such as Fantine’s *“I Dreamed a Dream”* and Valjean’s *“Bring Him Home”*, close-up shots allow the audience to perceive micro-expressions, vocal strain, and subtle shifts in facial movements, elements that are far less accessible in a theater setting. In this way, cinema does not replace theatrical emotion; rather, it redirects and intensifies it through visual intimacy. This aligns with Metz’s (1982) assertion that cinema generates a distinct mode of emotional perception through visual magnification.

The film’s visual design further supports this pathway. Its muted color palette, gritty realism, and controlled lighting do not merely reproduce stage spectacle on screen. Instead, these elements place musical performances within a coherent cinematic world, allowing the songs to emerge as part of the narrative environment rather than interrupting it. As Jie and Shen (2023) observe in their comparative analysis of Les Misérables, the film prioritizes performance over vocal perfection, employing speech-like delivery, rubato adjustments, and emotionally fragile singing to enhance cinematic realism, an approach distinct from the stage tradition’s fuller, more sustained vocal production. This integration of performance, imagery, and atmosphere reflects Gorbman’s (1980) argument that music and mise-en-scène coalesce to produce meaning.

From the perspective of the CASS model, *Les Misérables* achieves strong alignment across all three symbolic levels. At the surface level, vocal style, costume, and gesture are rendered with clarity and immediacy. At the structural level, the central redemption arc remains emotionally intelligible. At the deep level, themes such as sacrifice, suffering, and social justice are translated into forms that resonate with global audiences, extending beyond the original theatrical context. As a result, the adaptation succeeds not by replicating the stage version but by transforming stage language into a cinematic form that maintains symbolic coherence.

***The Phantom Lover (1995)***

As a supplementary Chinese reference rather than a focal case, *The Phantom Lover* helps illustrate certain features of Cinematic Translation, particularly the combination of stage-derived emotional intensity with cinematic framing. The film preserves the emotional immediacy often associated with stage singing, yet it does not rely solely on theatrical presentation. Instead, it uses close-up shots, low-key lighting, and carefully constructed visual spaces to reshape this immediacy into a more intimate cinematic experience. Leslie Cheung’s performance, in particular, mediates between theatrical expressivity and cinematic restraint, allowing the performance to remain emotionally legible without theatrical exaggeration.

This transformation is reinforced by the film’s visual design. Rather than simply recording a stage-like performance, the cinematography creates a stylised cinematic world through sharp contrasts of light and shadow, layered interior spaces, and symbolic set designs. These visual choices do more than establish atmosphere; they help translate the emotional and aesthetic intensity of theatrical performance into a film language that is both visually coherent and psychologically focused. Thus, the film does not merely preserve theatrical aesthetics but reworks them into a cinematic form.

However, *The Phantom Lover* also highlights the limits of Cinematic Translation. While the film achieves emotional and visual coherence for viewers familiar with its cultural and historical context, some of the cultural symbols and emotional codes become less legible outside that interpretive framework. This is particularly noticeable when culturally specific imagery is presented without adequate narrative mediation for non-Chinese audiences. This suggests that successful Cinematic Translation not only requires medium-specific reformulation but also depends on how accessible cultural symbols remain across diverse viewing contexts.

##### 4.1.1.2 Pathway 2: direct transplantation of stage language

***Jinsha (2023)***

*Jinsha* illustrates the pathway of Direct Transplantation, in which stage-derived performance conventions are carried over into film with relatively limited cinematic reformulation. The adaptation retains extended musical sequences, frontal staging, and stylised modes of gesture and movement that are effective in theatrical space but less fully adjusted to the visual logic of film. As a result, several musical scenes appear detached from the surrounding narrative, producing what focus group participants described as *“performance islands.”*

This problem is especially visible in the relationship between spectacle and narrative movement. The film makes extensive use of elaborate sets, digitally enhanced landscapes, and visually striking historical imagery. However, many musical passages are still filmed in ways that preserve stage blocking rather than exploit camera movement, shot variation or spatial reorientation. In practice, this means that visually rich scenes do not always generate stronger dramatic progression. As one participant noted, the imagery was impressive, but it did not make the story easier to follow or emotionally more engaging.

This pattern is significant in the context of the original Chinese musical theater film. In *Jinsha*, culturally specific elements such as costume design, ritualized choreography, and historical atmosphere are highly visible. These features contribute to surface-level symbolic clarity, especially for domestic viewers familiar with this visual and cultural vocabulary. Yet their prominence does not automatically produce structural coherence. Narrative compression weakens character development, and the emotional function of song is not always fully integrated into plot progression. At the deeper symbolic level, themes linked to historical memory and cultural inheritance remain present, but they are not consistently anchored in dramatic action. The case therefore, suggests that when theatrical elements are transferred to film without sufficient cinematic restructuring, cultural richness may remain visually impressive while losing narrative and affective force.

***Dear Evan Hansen (2021)***

*Dear Evan Hansen* provides an Anglo-American example of Direct Transplantation, although the issues it reveals differ from those seen in *Jinsha*. The film retains much of the stage musical’s narrative structure, song arrangement, and central performance style, especially through Ben Platt’s return from the stage production. However, these theatrical elements are transferred to the screen with limited adjustment to cinematic realism, creating tensions that the film does not fully resolve.

The central problem lies in the relationship between performance scale and visual framing. On stage, emotional intensity is sustained through live co-presence and theatrical convention. In the film, however, close framing and naturalistic settings place that same performance under a different set of expectations. Expressions and vocal delivery that carry strong emotional force in theater appear more exposed and less fully naturalized on screen. This is especially evident in scenes where the film remains visually restrained while asking the audience to accept highly stylised musical expression within an otherwise realist world.

This mismatch affects both narrative flow and emotional credibility. Rather than emerging organically from the cinematic environment, several musical numbers feel inserted into it. As a result, the film preserves recognizable elements of the stage version but does not consistently transform them into a screen language with its own coherence. The issue is not simply fidelity to the source, but the lack of formal mediation between theatrical performance and cinematic expression.

From the perspective of the CASS model, *Dear Evan Hansen* retains strong surface-level symbols. The songs remain familiar, and Platt’s star presence functions as an immediate point of recognition. At the structural level, however, themes of adolescent anxiety, isolation, and miscommunication lose part of their force because the emotional logic of stage performance is not fully reworked for film. At the deeper level, the adaptation remains tied to culturally specific codes of American youth experience, which may further restrict resonance beyond its primary interpretive context. The case, therefore, shows that Direct Transplantation can preserve the visible content of a musical while weakening its dramatic and communicative impact on screen.

##### 4.1.1.3 Pathway 3: fragmented reconfiguration of stage language

***The Long Night (2022)***

*The Long Night* represents the pathway of Fragmented Reconfiguration, in which stage-derived performance elements are not preserved as complete theatrical units, nor fully translated into a continuous cinematic form. Instead, they are broken apart and selectively incorporated into a predominantly cinematic structure. In this pathway, stage language functions less as a stable mode of presentation than as a source of expressive material that can be redistributed across the film.

This is most evident in the film’s non-linear narrative design. Rather than presenting performance in extended and self-contained sequences, the film intercuts heightened performative moments with shifts in time, mood, and visual perspective. The effect is not one of straightforward continuity, but of partial accumulation. For some viewers, this structure created a more active mode of engagement. Several focus group participants described the experience as *“puzzle-like,”* suggesting that fragmentation encouraged interpretation rather than simply causing confusion.

The same pattern appears in the treatment of movement and gesture. Instead of preserving performance as a complete musical spectacle, the film disperses stage-derived expression into shorter visual and affective units. Gesture, posture, and choreographic emphasis appear as intensified moments within cinematic flow rather than as separate performance blocks. One participant described these moments as “emotional punctuation,” which captures their function well. They do not suspend the narrative in the way that transplanted stage numbers often do. Instead, they punctuate it, redirecting attention and affect at key points.

From the perspective of the CASS model, *The Long Night* suggests that symbolic discontinuity does not always signal adaptive failure. When fragmentation is formally controlled and tied to narrative purpose, it can produce a distinct kind of aesthetic coherence. At the same time, this pathway appears more dependent on audience orientation than the other two. Participants who were more comfortable with non-linear form or with hybrid theatrical and cinematic codes were generally more receptive to it. Others found the structure distant or difficult to follow. This suggests that Fragmented Reconfiguration can be effective, but its success depends not only on formal design, but also on the interpretive expectations viewers bring to the film.

#### 4.1.2 Content analysis results: patterns across cases

Content analysis across the four cases reveals clear differences in the ways performance language is reorganized for the screen. Three dimensions proved particularly significant. These include the relationship between narrative compression and performance integration, the degree of alignment between musical expression and cinematic form, and the function of visual spectacle within narrative development.

##### 4.1.2.1 Narrative compression and performance integration

The cases show that narrative compression does not operate uniformly across adaptations. In *Les Misérables*, compression supports performance integration. By reducing subplots and tightening narrative focus, the film creates space for musical sequences that continue dramatic development rather than interrupt it. Songs remain embedded in the forward movement of the story and sustain emotional progression.

In *Jinsha*, however, compression simplifies the narrative without a corresponding transformation of performance form. Although the storyline becomes more streamlined, the musical sequences are not fully reworked for cinematic narration. As a result, they often function less as vehicles of dramatic development than as visually and aurally heightened insertions. This contrast suggests that narrative compression contributes to successful adaptation only when it is accompanied by a comparable reorganisation of performance language.

##### 4.1.2.2 Musical integration and cinematic alignment

A second pattern concerns the relationship between musical performance and cinematic form. *Les Misérables* achieves a relatively strong alignment between the two. Live singing, close framing, and sustained visual attention combine to produce a mode of expression that remains emotionally intense while also functioning coherently within the film’s visual language.

*Dear Evan Hansen* presents the opposite tendency. In this case, performance style is largely retained from the stage production, but it is placed within a more restrained and naturalistic cinematic environment. This produces a visible tension between stylised musical expression and realist screen form. The contrast between the two films supports Gorbman’s (1980) argument that musical performance in film must be reshaped in accordance with the expressive logic of cinema, rather than transferred directly from theatrical convention.

##### 4.1.2.3 Visual spectacle and narrative function

A third pattern concerns the relationship between visual spectacle and narrative function. In the Chinese cases, visual design is especially prominent, but its contribution to audience engagement varies depending on how closely it is tied to narrative development. Spectacle, in itself, does not guarantee stronger emotional or interpretive involvement.

In *Jinsha*, elaborate costumes, digitally enhanced settings, and highly stylised visual composition were frequently recognized by participants as aesthetically impressive. However, these elements were also often described as insufficiently connected to character development and story progression. The visual richness of the film therefore, enhanced surface-level attraction without consistently deepening narrative comprehension or emotional investment.

*The Long Night* presents a different pattern. Its visual style is comparatively more restrained, and its expressive force depends less on display than on selective emphasis. Combined with the film’s fragmented structure, this restraint allowed certain viewers to engage more actively with the unfolding narrative. In this case, visual design functioned less as ornament and more as a component of narrative rhythm and affective modulation.

Taken together, these cases suggest that the effectiveness of spectacle depends not on visual scale alone, but on the extent to which image, performance, and narrative are integrated. When spectacle remains largely decorative, it risks producing symbolic excess without corresponding emotional depth. When it is structurally embedded, it can contribute to a more coherent and engaging screen adaptation.

#### 4.1.3 Audience research results

##### 4.1.3.1 Participant demographics

In addition to age, participants also differed in linguistic background and cultural exposure (see Table 1). Most were based in China, while a small number were located overseas. Among the 27 participants, 24 reported bilingual competence in Chinese and English. Specifically, all 21 participants who were receiving or had received secondary or higher education (including the 3 teenagers and 18 young adults) were bilingual, as were 3 of the 6 middle-aged and older participants. However, only a limited number had substantial cross-cultural experience. This background is important for interpreting the reception data, because the study addresses performance language across cultural contexts, whereas the participant pool remained primarily China-based.

**TABLE 1:** Participant demographics, age distribution and general musical familiarity.

Age group	Range	Participants	Share	General musical familiarity
Teenagers	12–17	3	11.1%	Very limited exposure; generally had only heard of musicals.
Young adults	18–30	18	66.7%	Mixed familiarity; ranged from basic awareness to regular viewing and active interest.
Middle-aged adults	31–50	2	7.4%	Generally aware of musicals but less familiar overall; some expressed positive interest.
Older adults	Over 50	4	14.8%	Some occasional viewing, but overall familiarity remained limited.

The sample also showed variation in prior familiarity with musicals. Teenagers generally had only limited awareness of the genre, while young adults displayed the broadest range of familiarity, from minimal knowledge to regular viewing and active interest. The middle-aged and older groups were generally less familiar with musicals overall, although some participants expressed positive attitudes toward musical performance. These differences in prior familiarity are relevant to the interpretation of audience response, particularly in relation to emotional immersion, narrative clarity, and tolerance for stylised performance.

At the same time, the age distribution of the sample was uneven. Participants aged 18 –30 formed the largest subgroup, while the number of participants aged over 50 remained relatively small. For this reason, any age-related patterns identified in the later analysis should be understood as exploratory tendencies within this sample rather than as generalisable generational conclusions.

**FIGURE 2:**
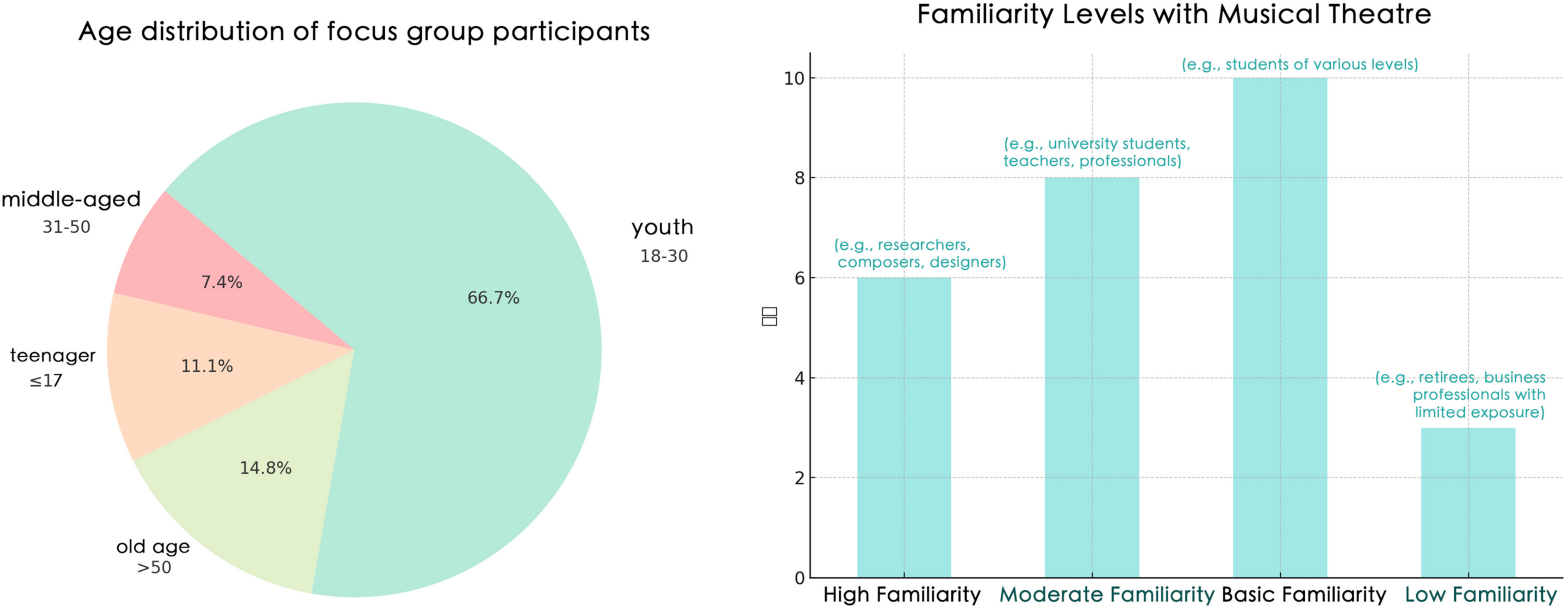
Age Distribution and general musical familiarity among focus group participants.

**FIGURE 3:**
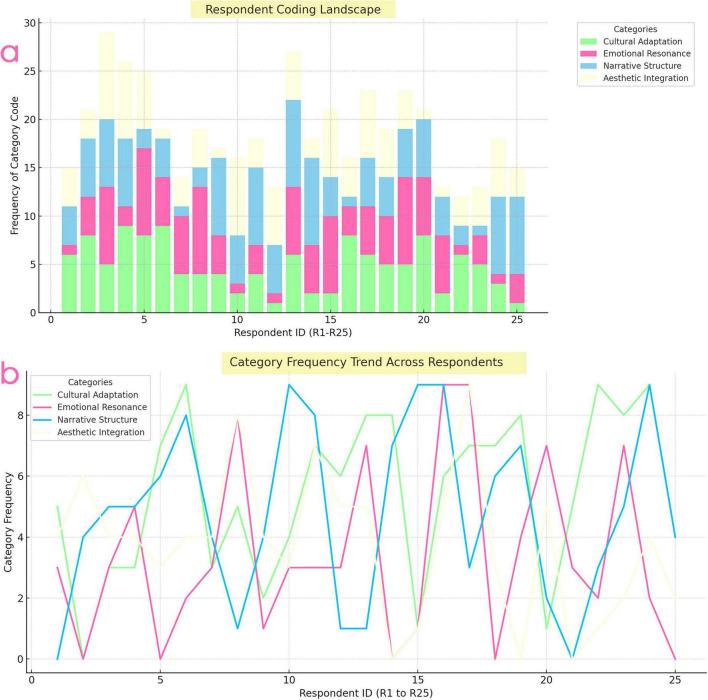
**(a)** Stacked bar chart labeled “respondent coding landscape” showing the frequency of four code categories (cultural adaptation, emotional resonance, narrative structure, aesthetic integration) across 25 respondents (R1–R25). **(b)** Line chart titled “category frequency trend across respondents” plotting the same four categories’ frequency trends per respondent.

Figure 2 presents the overall distribution of age and general musical familiarity among the participants. Figure 3 summarizes the coding landscape across respondents and shows the relative frequency of the main thematic categories identified in the focus group data.

#### 4.1.4 Integrated findings: three pathways and audience response

The integration of case analysis, content analysis, and audience research identified three distinct pathways of performance language transformation, each associated with a different pattern of audience response. Taken together, the findings suggest that audience engagement depends not simply on the presence of musical performance or cultural symbolism, but on the extent to which performance language is reorganized into a coherent cinematic form.

##### 4.1.4.1 Quantitative evaluation

Participants rated the selected excerpts across four dimensions using five-point Likert scales: emotional immersion, cultural adaptability, aesthetic satisfaction, and narrative clarity. Table 2 presents the mean scores for the Chinese and Anglo-American cases included in this study.

**TABLE 2:** Average audience ratings of chinese and anglo-american musical adaptations across four evaluation dimensions.

Evaluation dimension	Anglo-american mean	Chinese mean	Significant difference (*p* < 0.05)
Emotional immersion	4.5	2.9	✓
Cultural adaptability	4.6	2.7	✓
Aesthetic satisfaction	4.1	3.4	×
Narrative clarity	4.3	2.8	✓

Based on paired-sample *t*-tests comparing mean ratings of Chinese and Anglo-American cases within the same participant sample (*N* = 27). Differences with *p* < 0.05 are marked “✓.” Actual *p*-values: Emotional Immersion *p* = 0.012, Cultural Adaptability *p* = 0.008, Narrative Clarity *p* = 0.021.

**FIGURE 4:**
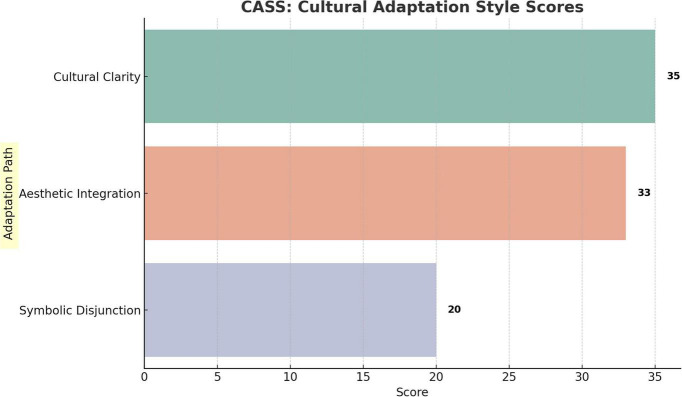
CASS model evaluation scores across three adaptation pathways.

**FIGURE 5:**
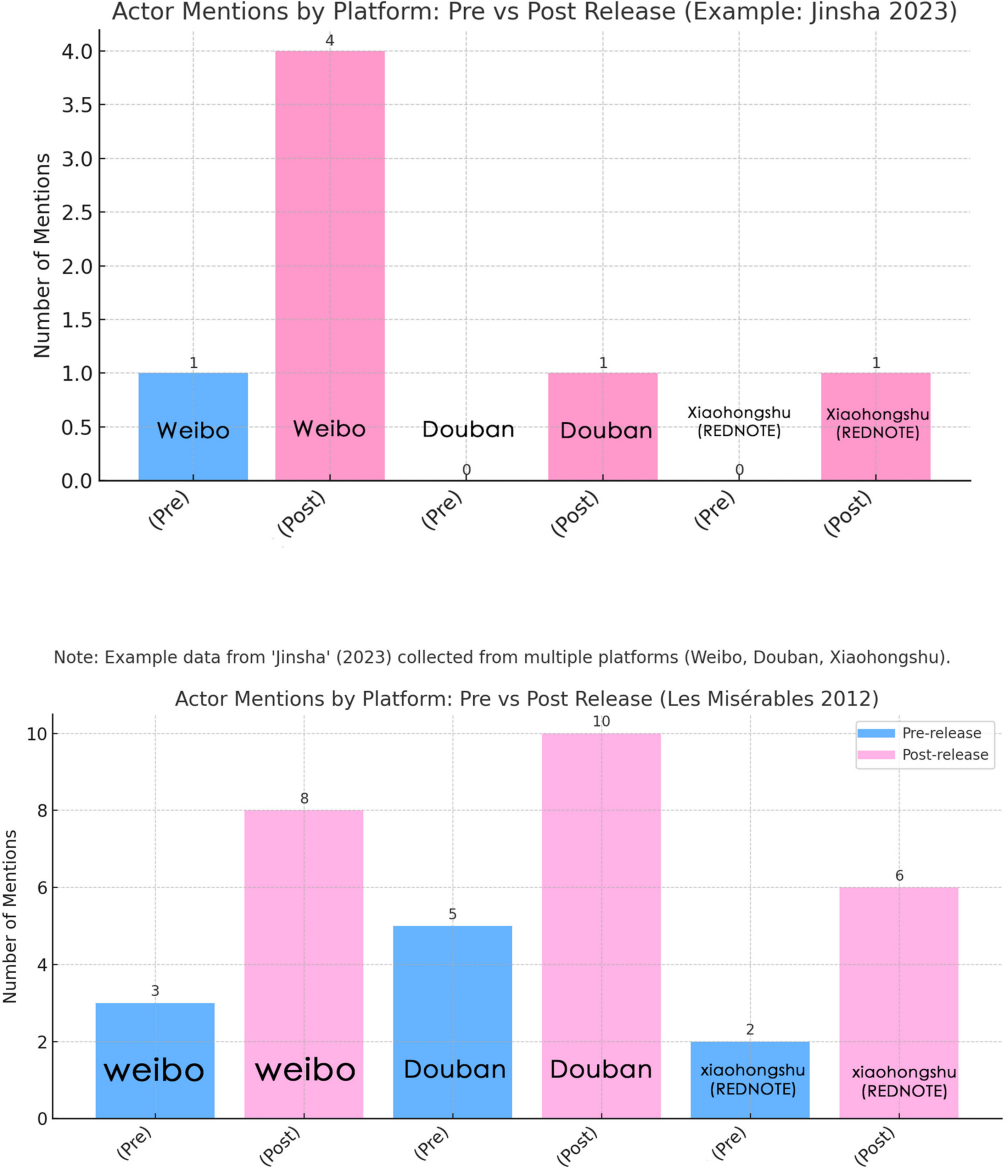
Actor keyword mentions by platform and time (Jinsha, 2023; Les Misérables, 2012).

**FIGURE 6:**
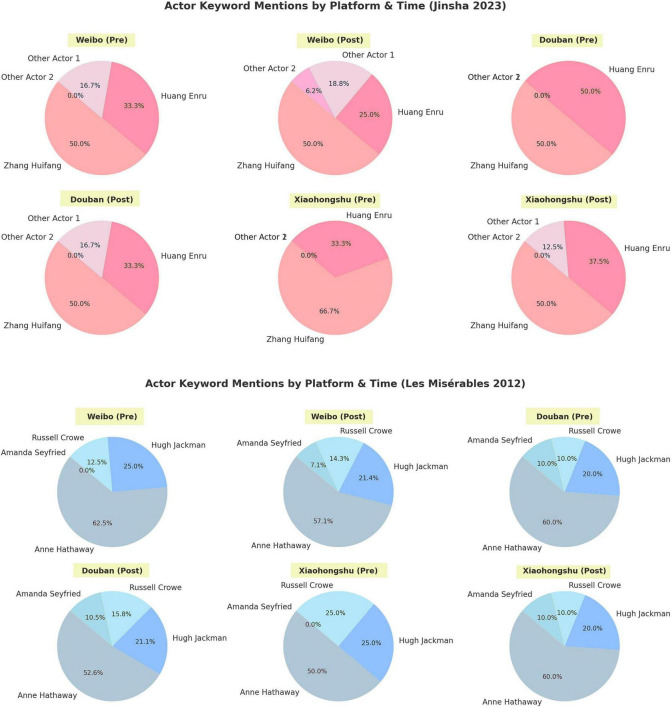
Actor mentions by platform before and after release (Jinsha, 2023; Les Misérables, 2012).

Within this sample of 27 participants, the Anglo-American cases received higher mean scores in emotional immersion, cultural adaptability, and narrative clarity (see Figures 4–6). Aesthetic satisfaction did not differ significantly. This suggests that visual richness alone was not sufficient to generate stronger narrative understanding or emotional involvement. Put differently, the Chinese cases were often recognized as visually impressive, but this did not consistently translate into greater interpretive clarity or affective engagement.

These results should nevertheless be interpreted cautiously. The participant pool was relatively small, unevenly distributed across age groups, and composed primarily of China-based viewers. In addition, participants varied considerably in their prior familiarity with musicals. For this reason, the quantitative comparison is best understood as indicating response patterns within this sample rather than as a definitive generalization about Chinese and Anglo-American musical theater film adaptation more broadly.

##### 4.1.4.2 CASS model scoring

To relate audience response more directly to the CASS model, the focus group data were further organized into three interpretive categories: Cultural Clarity, Aesthetic Integration, and Symbolic Disjunction. These scores were derived from thematic recurrence and interpretive cohesion across the coded participant responses. They should therefore be understood as interpretive summary scores rather than purely statistical measurements. Higher scores indicate that a particular response pattern appeared more consistently across the five focus groups.

These scores represent the total number of coded instances associated with each interpretive category across the focus-group transcripts (see Table 3). Because a single participant response could receive multiple codes, the scores indicate relative thematic prominence rather than mutually exclusive participant counts.

**TABLE 3:** CASS-based audience response scores.

CASS dimension	Score	Interpretation
Cultural clarity	35	Cultural symbols were perceived as legible and contributed to audience understanding of the work’s background and meaning.
Aesthetic integration	33	Visual design and musical expression were generally perceived as working together, although not always with complete stylistic coherence.
Symbolic disjunction	20	Symbolic elements were recognized, but their relationship to narrative and emotional development was experienced as weak or unstable.

These scores suggest that audience response was shaped not only by the visibility of symbolic elements on screen, but also by the extent to which those elements were integrated across expressive levels. Cultural Clarity and Aesthetic Integration were most strongly associated with the pathway of Cinematic Translation, most clearly represented by *Les Misérables.* Symbolic Disjunction was most strongly associated with Direct Transplantation, as seen in *Jinsha* and *Dear Evan Hansen*. Fragmented Reconfiguration, represented by *The Long Night*, occupied a more variable position. Its reception was less uniform and appeared to depend more heavily on audience expectations, familiarity with non-linear form, and willingness to engage with hybrid theatrical and cinematic codes.

##### 4.1.4.3 Qualitative illustrations

The qualitative data further support the distinctions identified in the case analysis, content analysis, and audience ratings. Coding of the focus group transcripts revealed three recurring interpretive tendencies that broadly corresponded to the three pathways of performance language transformation.

##### 4.1.4.4 Responses associated with cultural clarity

Participants who responded positively to Cinematic Translation consistently emphasized emotional accessibility and interpretive legibility. One participant remarked, *“Stage musicals hit you emotionally; films zoom in to dissect every feeling.”* Another commented that *Les Misérables “made me understand the characters’ struggles in a way I never did watching stage productions.”* These responses suggest that Cinematic Translation was most effective when it preserved the emotional intensity of theatrical performance while rendering it more readable through cinematic framing, visual intimacy, and narrative continuity.

##### 4.1.4.5 Responses associated with aesthetic integration

Participants who responded positively to Fragmented Reconfiguration often described the viewing experience as more active and interpretive. In relation to *The Long Night*, one participant observed, *“Non-linear structure is like a puzzle. When it fits, it hits.”* Another said that the film *“trusts you to put the pieces together.”* Such responses indicate that, for some viewers, fragmentation did not weaken engagement but instead encouraged a more participatory mode of interpretation.

At the same time, this response pattern was not shared across the whole sample. While some viewers found the fragmented structure stimulating and aesthetically coherent, others experienced it as distance, interruption, or reduced clarity. This suggests that the effectiveness of Aesthetic Integration in this pathway was conditional rather than uniform, depending in part on viewers’ familiarity with non-linear storytelling and hybrid performance forms.

##### 4.1.4.6 Responses associated with symbolic disjunction

Participants responding to Direct Transplantation frequently described a gap between visible performance elements and meaningful narrative involvement. In relation to *Jinsha*, one participant commented, *“The Han costume took me right into Jinsha, but I still didn’t get the story.”* A similar response emerged in relation to *Dear Evan Hansen*: *“I could see Ben Platt was giving everything, but it felt like watching someone act rather than being inside the moment.”* Although the two films differ in cultural context and performance style, the audience response revealed a comparable pattern. In both cases, viewers were able to recognize the presence of strong surface-level elements, including costume, vocal delivery and performer intensity, yet these elements did not consistently translate into deeper emotional immersion or narrative coherence.

These responses suggest that symbolic visibility alone is not sufficient to sustain audience engagement. When stage-derived performance conventions are transferred to film without adequate cinematic reorganization, symbolic richness remains perceptible but loses part of its dramatic force. What the audience recognizes visually or performatively is not always what the film succeeds in integrating narratively or affectively.

Taken together, the quantitative and qualitative findings support the identification of three pathways of performance language transformation and clarify how each pathway shapes audience response in different ways. More broadly, they suggest that the adaptation of Chinese original musicals into musical theater film depends not simply on the preservation of cultural symbols or theatrical performance, but on the extent to which these elements are cinematically restructured and embedded within a coherent narrative and emotional framework.

### 4.2 Discussion

#### 4.2.1 The three pathways: theoretical implications

The identification of three pathways refines the CASS model’s application to musical theater film adaptation. While surface, structural, and deep cultural symbols appear across all four cases, they operate differently. What varies is not the amount of symbolic material retained, but how well symbolic visibility, narrative organization, and emotional legibility remain connected after adaptation.

In Cinematic Translation, surface elements are cinematically reformulated while remaining aligned with structural and deep symbolic levels. Performance language retains emotional force because the three levels cohere.

In Direct Transplantation, stage conventions remain visibly intact but lose narrative and affective coherence. Surface symbols stay legible, yet their dramatic function weakens; visual preservation does not guarantee narrative or emotional continuity.

In Fragmented Reconfiguration, stage-derived elements are broken apart and redistributed within a cinematic structure, producing a less stable relationship among symbolic levels. Fragmentation can generate aesthetic engagement and active interpretation, but may also reduce accessibility. Symbolic discontinuity is not automatically a failure; its effectiveness depends on formal control and narrative embedding.

Together, these pathways extend adaptation theory beyond fidelity-based evaluation. As Hutcheon (2006) argues, adaptation depends on re-encoding rather than reproduction. The issue is not whether stage language is preserved or transformed, but whether performance elements keep symbolic meaning, narrative function, and audience engagement in productive relation. The CASS model thus helps not only to identify symbolic content, but also to show how different configurations of performance language shape adaptation’s possibilities and limits. This is especially important for Chinese original musicals, where the challenge is to restructure cultural richness into a cinematically coherent and emotionally effective form.

#### 4.2.2 Age-related tendencies in performance language reception

The focus group data suggest some age-related differences in responses to the three pathways of performance language transformation, but these patterns should be interpreted with caution. The sample was unevenly distributed across age groups. Participants aged 18–30 formed the largest subgroup, while the number of middle-aged and older participants remained limited. In particular, the middle-aged group included only two participants, and the group aged over 50 included only four. As a result, the present findings can reflect only partial and preliminary tendencies among middle-aged and older viewers, rather than robust or generalisable age-based conclusions.

Younger participants were generally more receptive to Cinematic Translation. Many described this pathway as more *“authentic”* or emotionally convincing on screen. Their responses suggest a preference for performance styles that align more closely with cinematic realism, even when the source material is theatrical. In this context, adaptations that retained visible stage conventions without substantial cinematic reformulation were sometimes perceived as artificial, overly staged, or emotionally distancing.

By contrast, the small number of middle-aged and older participants appeared somewhat more accepting of Direct Transplantation. In several responses, the retention of theatrical conventions was interpreted less as a weakness than as a sign of continuity with stage performance. For these viewers, visible theatricality could still carry aesthetic value. However, because these age groups were represented by only a few participants, this tendency should be treated as suggestive rather than conclusive.

Responses to Fragmented Reconfiguration were the most variable. Reception in this case seemed to depend less on age alone than on audience orientation toward non-linear storytelling and hybrid performance forms. Participants who were more comfortable with experimental narrative structures were generally more open to fragmentation, while others found it difficult to follow. This suggests that performance language reception is shaped not only by age but also by prior viewing habits, aesthetic expectations, and familiarity with both theatrical and cinematic conventions.

Taken together, these findings indicate that differences in audience response are better understood as the result of interacting factors rather than fixed generational divisions. Age may influence reception, but in the present study, its role can only be discussed in terms of limited and preliminary tendencies, especially for the middle-aged and older groups.

#### 4.2.3 Celebrity culture and performance language mediation: insights from Chinese social media

Analysis of social media data from Douban, Weibo and Xiaohongshu further illustrates how celebrity culture interacts with performance language mediation. Figures 5, 6 show actor mention patterns for *Jinsha (2023)* and *Les Misérables (2012)* across different platforms and release periods. These data do not provide a fully symmetrical cross-cultural comparison, since the platforms analyzed are primarily Chinese-language platforms. They are nevertheless useful for showing how celebrity visibility shaped audience attention within the reception context available to this study.

In *Jinsha*, audience attention was concentrated on a small number of performers. Zhang Huifang accounted for nearly half of all mentions, while Huang Enru showed a marked increase after release, especially on Weibo and Xiaohongshu. Other cast members attracted much less discussion. This pattern is consistent with a headliner-driven promotional structure and reflects the broader visibility logic of China’s idol economy, in which star recognition often functions as the main entry point for audience engagement.

By contrast, *Les Misérables* generated a more distributed pattern of attention. Anne Hathaway received the highest proportion of mentions, but Hugh Jackman, Russell Crowe, and Amanda Seyfried also maintained substantial visibility. The post-release increase in discussion was spread more evenly across performers and platforms. This suggests a broader ensemble-based attention structure, supported not only by star status, but also by the film’s wider media circulation and awards discourse.

From the perspective of the CASS model, celebrity culture operates across multiple symbolic levels. At the surface level, star names function as immediate recognition cues that attract attention and shape first impressions. At the structural level, casting patterns influence how attention is distributed across characters and narrative lines. At the deep level, celebrity persona can shape interpretive expectations by redirecting audience focus from character and story toward public image and prior associations.

These patterns are important for understanding performance language mediation. When celebrity presence supports performance, characterisation, and narrative structure, it can strengthen engagement. When it substitutes for them, symbolic disjunction becomes more likely. In such cases, surface recognition remains strong, but structural and affective integration weakens. As Liu (2024) suggests, celebrity culture can increase visibility, but it cannot compensate for insufficient narrative coherence or for the incomplete cinematic mediation of stage-derived performance.

#### 4.2.4 Implications for practice

The findings carry several practical implications for the adaptation of original Chinese musicals into musical theater film, especially in a context where the genre is still developing and its cinematic language remains relatively unsettled.

First, successful adaptation requires more than preserving memorable stage elements. It requires the deliberate redesign of performance language for the screen. The evidence from *Les Misérables* shows that live singing, close framing, and carefully controlled visual environments can translate theatrical intensity into a form that remains emotionally legible in film. What matters is not simple retention, but the cinematic integration of song, gesture, and dramatic focus.

Second, the findings suggest that Direct Transplantation carries clear risks. When stage conventions are transferred to film without sufficient formal adjustment, musical numbers may become detached from narrative progression and emotional development. In the present study, this often resulted in what participants described as *“performance islands.”* For Chinese original musicals in particular, this risk becomes more pronounced when visual spectacle is prioritized while performance language remains insufficiently adapted to cinematic storytelling.

Third, Fragmented Reconfiguration offers an alternative path, but its effectiveness depends on a clear aesthetic rationale. Fragmentation can generate a distinctive form of engagement when it is meaningfully embedded in narrative design and supported by coherent visual and affective logic. However, when fragmentation appears to result from incomplete adaptation rather than deliberate formal strategy, it is more likely to be experienced as confusion or distance.

Fourth, celebrity casting should be treated as a supporting strategy rather than a substitute for adaptation work. Star power can expand visibility and attract audience attention, but it cannot compensate for weak narrative integration or unresolved tension between stage-derived performance and cinematic form. When celebrity persona dominates audience perception, the adaptation risks becoming organized around surface recognition rather than around dramatic coherence.

Taken together, these implications suggest that the future adaptation of Chinese original musicals into musical theater film depends not only on cultural richness or visual ambition, but on the creation of a stable and effective screen language for musical performance. For filmmakers, this means closer coordination between performance style, camera design, editing rhythm, and narrative structure. For performers, it means adjusting expressive scale and intensity for film without losing emotional credibility. More broadly, for the development of future Chinese stories in musical theater film form, the central task is not simply how to preserve theatrical identity, but how to transform it into a cinematically coherent and emotionally engaging mode of expression.

#### 4.2.5 Limitations, critical reflection, and future research

This study has several limitations. First, the participant sample is small and unevenly distributed across age groups, limiting generalisability. Age-related patterns should therefore be treated as preliminary. Second, the participant pool is primarily China-based, so the reception data reflect China-based perspectives rather than a fully balanced intercultural comparison. Third, the study focuses on a small number of cases, which allows comparative depth but cannot represent the full range of musical theater film adaptation practices. The Anglo-American cases should not be taken as representative of all Anglo-American musical traditions. Fourth, the cross-sectional design captures audience response at a single point in time, not changes through repeated viewing or longer-term cultural learning.

These limitations point to several future directions. Future studies can integrate Liu’s (2024) framework of *“center and decentering”* in cross-cultural adaptation, where each adaptation’s center inherits the decentering of its predecessor, and Xu’s (2022) practice-based approach to vocal characterization, including timbre selection, articulatory adjustment, and body-voice integration, to examine how ideological tensions and performance choices jointly shape audience reception in Chinese musical film adaptations. Further studies could expand and balance the sample, include more varied linguistic and intercultural backgrounds, and incorporate a wider range of cases, including other Asian musical traditions. The most important direction lies in refining the pathways through which Chinese original musicals can be adapted into film, examining how Chinese stories, cultural symbols, and performance traditions can be cinematically restructured without losing emotional and cultural specificity. Longitudinal designs and targeted comparative experiments would also help clarify how adaptation choices affect audience response over time.

## 5 Conclusion

This study examined how musical theater is adapted into film in Chinese and Anglo-American contexts, with particular attention to the mediation of performance language from stage to screen. Drawing on the CASS model and combining case analysis, content analysis, focus groups, and questionnaires, the study explored how performance language is retained, reshaped, or disrupted in adaptation. The findings show that successful adaptation depends less on preserving stage conventions than on reorganizing them into cinematically meaningful forms.

The analysis identified three pathways of stage-to-screen transformation. Cinematic Translation (*Les Misérables, 2012*) reworks stage elements through cinematic devices, producing the strongest audience engagement in emotional immersion and narrative clarity. Direct Transplantation (*Jinsha, 2023; Dear Evan Hansen, 2021*) retains visible stage conventions but weakens narrative and affective coherence. Fragmented Reconfiguration (*The Long Night, 2022*) breaks apart stage-derived elements within a cinematic structure, yielding mixed responses that depend on viewers’ interpretive expectations.

These pathways answer the research questions in several ways. They show that stage language is transformed through different strategies of cinematic reformulation, that these strategies shape audience response differently, and that celebrity visibility cannot compensate for weak integration between performance, narrative, and screen form.

The study also found age-related tendencies, though these should be interpreted cautiously. Within this sample, younger participants were more receptive to Cinematic Translation, while some middle-aged and older participants were more tolerant of visible theatrical conventions. Responses to Fragmented Reconfiguration were especially variable, suggesting that audience orientation and familiarity with non-linear forms may matter as much as age.

Theoretically, this study shifts adaptation research from fidelity to performance language mediation, showing adaptation as the reorganization of embodied and audiovisual performance across media. The CASS model proves useful for analyzing how surface, structural, and deep symbolic levels cohere or diverge. The three pathways refine the model by showing that symbolic levels are configured differently depending on adaptation strategy.

Empirically, the study provides audience-based evidence to a field that has often remained theoretical. The findings indicate that retaining visible symbols is not enough; audience engagement depends on whether those symbols are connected to coherent narrative and affective structures.

The study also invites critical reflection on Chinese musical theater film adaptation. The weaker reception of some Chinese cases should not be reduced to a simple Anglo-American versus Chinese opposition. Rather, the adaptation of Chinese original musicals is still taking shape within a developing industrial, aesthetic, and audience environment. What appears as symbolic disjunction may signal a field in transition, searching for forms that can carry Chinese stories effectively onto the screen (Zheng, 2021).

Several limitations are acknowledged. The participant sample was small and unevenly distributed across age groups, limiting generalization. The audience pool was primarily China-based, so reception data reflect primarily China-based perspectives. The case selection does not represent the full range of adaptations. The celebrity culture discussion drew partly on Chinese-language platform data, making cross-cultural comparison not fully symmetrical. Finally, the study captures audience response at a single point in time.

These limitations point to future research directions. Further studies could expand and balance the sample, include more varied linguistic and intercultural backgrounds, and incorporate a wider range of cases, including other Asian musical traditions. The most important direction lies in refining the pathways through which Chinese original musicals can be adapted into film—examining which source material is most suitable, how songs can be cinematically motivated, and how film language can exceed rather than merely reproduce theatrical presentation.

In practical terms, successful adaptation requires more than visual richness or celebrity appeal. Cultural symbols are most effective when embedded within coherent dramaturgical and audiovisual structures. For filmmakers, decisions about song performance, camera movement, framing, editing, and narrative progression must work together. For performers, adaptation involves adjusting expressive scale without losing emotional credibility. For Chinese original musicals, the central challenge is not only displaying cultural specificity but making it dramatically persuasive and cinematically meaningful.

Overall, this study argues that musical adaptation is a negotiation among media forms, performance traditions, and audience expectations. Its central conclusion is that successful adaptation depends on effective mediation of performance language rather than fidelity alone. By identifying three pathways and examining audience responses, the study provides a framework for understanding why some adaptations achieve resonance while others produce disjunction. More importantly, it suggests that the future of Chinese original musical theater film lies not in copying existing models but in developing a stable cinematic language capable of carrying Chinese stories with emotional force and formal coherence.

## Data Availability

The original contributions presented in the study are included in the article/supplementary material, further inquiries can be directed to the corresponding author.
